# Radiomics based of deep medullary veins on susceptibility-weighted imaging in infants: predicting the severity of brain injury of neonates with perinatal asphyxia

**DOI:** 10.1186/s40001-022-00954-y

**Published:** 2023-01-06

**Authors:** Xiamei Zhuang, Huashan Lin, Junwei Li, Yan Yin, Xiao Dong, Ke Jin

**Affiliations:** 1 Department of Radiology, Hunan Children’s Hospital, 86 Ziyuan Road, Yuhua District, Changsha, China; 2 Department of Pharmaceutical Diagnosis, GE Healthcare, Changsha, 410005 China

**Keywords:** Magnetic resonance imaging, Deep medullary veins, Hypoxic-ischemic encephalopathy, Radiomics, Neonatal

## Abstract

**Objective:**

This study aimed to apply radiomics analysis of the change of deep medullary veins (DMV) on susceptibility-weighted imaging (SWI), and to distinguish mild hypoxic-ischemic encephalopathy (HIE) from moderate-to-severe HIE in neonates.

**Methods:**

A total of 190 neonates with HIE (24 mild HIE and 166 moderate-to-severe HIE) were included in this study. All of them were born at 37 gestational weeks or later. The DMVs were manually included in the regions of interest (ROI). For the purpose of identifying optimal radiomics features and to construct Rad-scores, 1316 features were extracted. LASSO regression was used to identify the optimal radiomics features. Using the Red-score and the clinical independent factor, a nomogram was constructed. In order to evaluate the performance of the different models, receiver operating characteristic (ROC) curve analysis was applied. Decision curve analysis (DCA) was implemented to evaluate the clinical utility.

**Results:**

A total of 15 potential predictors were selected and contributed to Red-score construction. Compared with the radiomics model, the nomogram combined model incorporating Red-score and urea nitrogen did not better distinguish between the mild HIE and moderate-to-severe HIE group. For the training cohort, the AUC of the radiomics model and the combined nomogram model was 0.84 and 0.84. For the validation cohort, the AUC of the radiomics model and the combined nomogram model was 0.80 and 0.79, respectively. The addition of clinical characteristics to the nomogram failed to distinguish mild HIE from moderate-to-severe HIE group.

**Conclusion:**

We developed a radiomics model and combined nomogram model as an indicator to distinguish mild HIE from moderate-to-severe HIE group.

**Supplementary Information:**

The online version contains supplementary material available at 10.1186/s40001-022-00954-y.

## Introduction

A patient with hypoxic-ischemic encephalopathy (HIE) may suffer from neurological problems as a result of neonatal asphyxia [1]. Globally, it causes 1 to 8 neonatal deaths per 1000 live births, making it one of the leading causes of neonatal morbidity and mortality [2]. Brain injuries caused by HIE lead to a serious condition, rapid progress and poor prognosis. Some patients can experience varying degrees of neurological sequelae. Sarnat scores are commonly used to evaluate neonatal HIE severity. Based on the clinical signs and electroencephalograms, according to Sarnat criteria, HIE can be mild (stage 1), moderate (stage 2), or severe (stage 3) [3]. HIE can be more effectively treated and predicted over the long term if the scope and severity of the disease are clarified and the diagnosis staged [4].

Although the Sarnat criteria was used to evaluate neonatal asphyxia clinically, the Sarnat score is subjective and easy to be impacted and affected by non-asphyxia factors. HIE is most commonly imaged with magnetic resonance imaging (MRI). There are, however, variations in the imaging patterns of HIE injury depending on the severity, the gestational age, the duration of the injury, and when the imaging is performed. Furthermore, in conventional T1WI and T2WI sequences, infants with unmyelinated brain structures and high-water content have difficulties discriminating pathologic signals and evaluating brain MRIs. In clinical practice, susceptibility-weighted imaging (SWI) is increasingly applied to HIE. The SWI is a high-resolution, 3D gradient echo imaging sequence, which uses the difference of magnetic sensitivity between tissues to display the paramagnetic properties of blood products through phase post-processing. Therefore, it is more sensitive to the detection of intravenous deoxyhemoglobin and extravascular blood products. HIE can cause contracture of small blood vessels in the brain and increase vascular permeability. Many factors cause vascular rupture and bleeding. Hypoxia and ischemia of brain tissue can cause congestion of cerebral veins and increase venous pressure, which is easy to cause dilation of deep cerebral veins in varying degrees [5]. It has been reported that DMV prominence is associated with both sinovenous thrombosis and white matter lesions (WM) in infants [6]. There have been few objective methods used to quantify DMV, even though it is thought to be a pathologic change observed during SWI.

MRI is an indispensable imaging modality for various clinical conditions, but it may only provide limited information due to human perception limitations. Radiomics is the extraction, analysis, and mining of medical data from digital medical images using high-throughput computational methods [7]. By selecting features from these data, biomarkers can be constructed for disease prediction and diagnosis. It is possible to obtain potentially valuable information through radiomics beyond the limitations of human analysis [8]. Numerous studies have investigated radiomics' clinical applicability. In various cancers, radiomics features have been shown to be useful imaging predictors of diagnosis, treatment response, prediction, and prognosis [9–11], and some studies use texture analysis to evaluating ischemic changes in neonates [12, 13]. There has been no previous data assessing Rad-score and combined nomogram model for HIE in neonates predicting the severity of brain injury. In this study, we developed and validated a combined nomogram model that extracted radiomics features from SWI to determine the severity of brain injury in neonates with HIE.

## Materials and methods

### Patients and data collection

This study was retrospectively approved by the medical ethics committee of the XX Children’s hospital. This retrospective study was approved by the institutional review board. Informed consent was waived due to the retrospective analysis of anonymized data.

In order to identify neonates with HIE or perinatal asphyxia, the neonatology department database was reviewed between January 2018 and April 2022. The inclusion criteria were as follows: (1) neonates with 37 weeks or later underwent MRI scan including SWI; (2) data of demographics, clinical information, and laboratory values were available. Exclusion criteria were as follows: (1) premature infants (gestational age $<$ 37 weeks), (2) on an MRI, infants with motion artifacts, and (3) infants with metabolism disease.

### MRI imaging acquisition

All patients underwent brain MRI scan, including SWI scan, preoperatively. All brain MRI scans were performed in our hospital on 3.0 T MRI scanner (MAGNETOM Skyra, Siemens or MAGNETOM Prisma, Siemens) with an eight-channel head coil using the same MR parameters. Axial SWI was used for extraction of radiomics features, the parameters for SWI were field-of-view 200 $\times$ 90.6 mm; voxel size 0.8 $\times$ 0.8 $\times$ 2.0 mm; slice thickness 2.0 mm; TR 27.0 ms; and TE 20.0 ms. In the institutional picture archiving and communication system (PACS), the MR images of the enrolled patients were exported in Digital Imaging and Communication in Medicine (DICOM) format and then converted to the NIFTI format using AK software (Artificial Intelligence Kit v.3.1.0.A, GE Healthcare).

### Image preprocessing

To remove potential differences between MR images acquired from two different scanners, we needed to preprocess the images before segmentation and feature extraction. We used AK software (Artificial Intelligence Kit v.3.1.0.A, GE Healthcare) to perform this procedure. The process is presented extendedly in Additional file 1: S1.

### Image segmentation and radiomics feature extraction

MR images were moved to a 3D slicer software for segmentation and saved for subsequent radiomics feature extraction. Additional file 2: S2 provide more details.

### Reproducibility

Radiomics reproducibility was evaluated intra- and inter-observer. Two observers performed the ROI analysis. 60 patients were randomly selected and delineated twice by observer 1 to ensure intra- and inter-observer reproducibility, with the same procedure and delineation conducted once by observer 2 to calculate the inter-observer ICCs. Generally, an ICC $>$ 0.75 is indicative of good agreement. The rest of the delineation was completed by observer 1.

### Feature selection and model construction

First, using a ratio of 7:3, patients were randomly divided into two cohorts: training cohort and validation cohort. Clinical features from the univariate analysis (*P* $<$ 0.05) were carried forward into the multivariate regression analysis. Features with *P* $<$ 0.05 in multivariate regression analysis were included in the clinical model.

It is important to understand that some features contribute to the positive performance of classification, while others may add noise [14]. In our radiomics model, the minimum redundancy maximum relevance (mRMR) was used to eliminate redundant and irrelevant features and retain those that were most predictive. Least absolute shrinkage and selection operator (LASSO) was conducted to select effective and predictable features for high-dimensional low-sample size data with collinearity problems. Features with non-zero coefficients were chosen based on tenfold cross-validation. The most predictive radiomics features were selected after the number of features was determined. By summing the selected features, weighted by coefficients, the Rad-score was calculated.

**Fig. 1:**
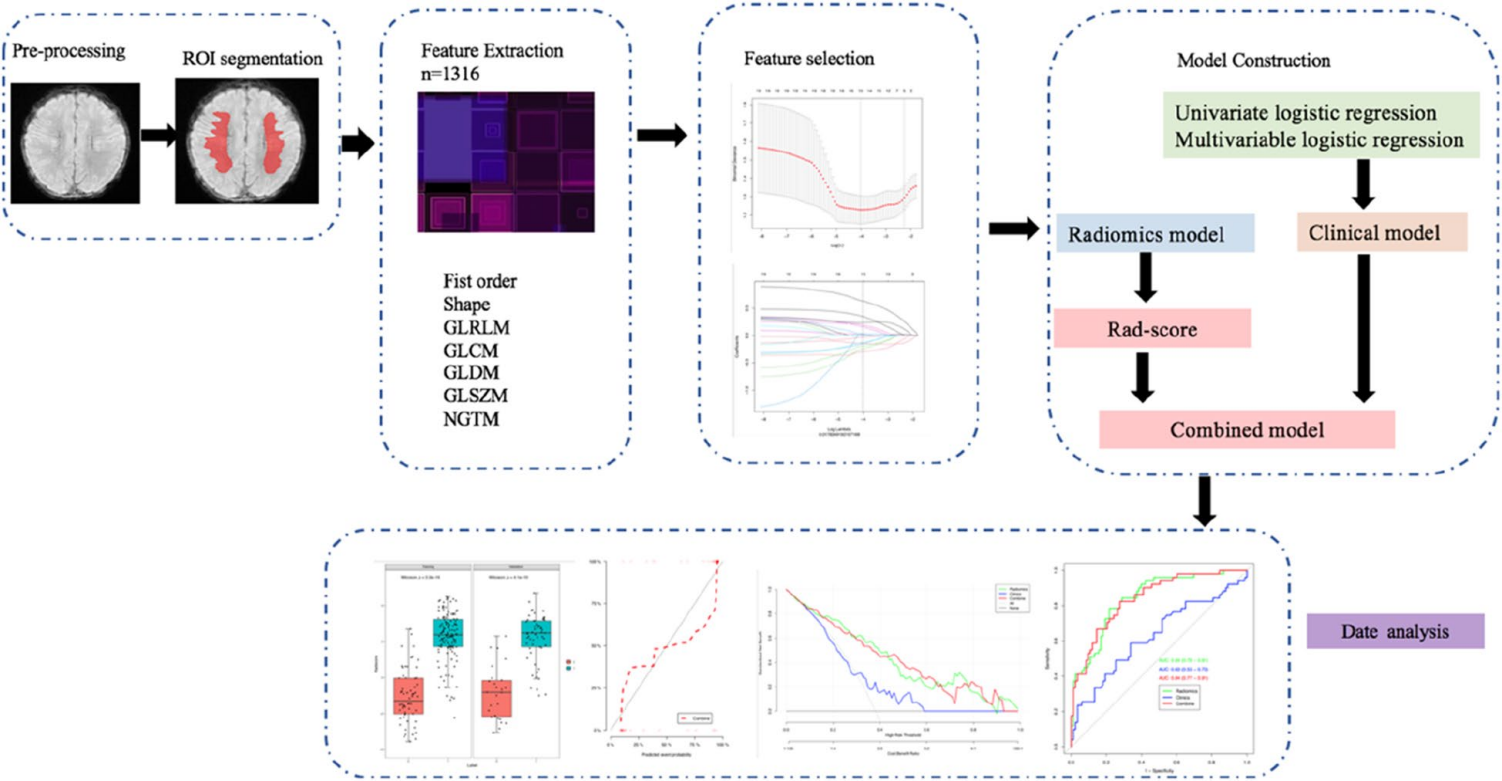
Workflow of this study. Workflow can be divided into five parts: image acquisition and ROI segmentation, feature extraction, feature selection, model construction, data analysis

A combination of the clinical signatures from the clinical model and Rad-score was used to develop the combined model with multivariate logistic regression. Figure 1 illustrates the workflow of the radiomics analysis.

### Model evaluation and validation

Based on receiver operating characteristic (ROC) curve and area under the curve (AUC) analyses, the diagnostic efficacy of different models was analyzed in the training cohort and validation cohort. In order to test the difference between ROC curves, the Delong test was used. In the training cohort and validation cohort, different predictive models were calibrated and evaluated. Calibration curves were evaluated using the Hosmer–Lemeshow test. A decision curve analysis (DCA) was used to evaluate the clinical value of different models.

### Statistical analysis

All statistical analysis used SPSS software (https://www.ibm.com, Version 26.0) and R software (https://www.Rproject.org, Version 4.1.0). The quantitative data were compared using Student’s t-test and Wilcoxon test. The categorical data were compared using the χ^2^ test. For the analysis of the mRMR, the "mRMRe" package was used. To execute LASSO, we used the "glmnet" package. The ROC curves were plotted using the "pROC" package. All statistical tests were two sided, with 0.05 set as the statistical significance.

## Results

### Clinical characteristics

This study included 190 neonates with HIE, including 24 with mild HIE and 166 with moderate-to-severe HIE. At a ratio of 7:3, each patient was randomly assigned to either the training cohort (*n* = 134) or the validation cohort (*n* = 56). The details of clinical characteristics and a comparison between mild group and moderate-to-severe group are presented in Table 1. It was found that significant differences existed between the groups in patients’ laboratory markers (urea nitrogen) and blood gas analysis (HCO^3−^, PH, base excess (BE)). There was no statistical difference between the training cohort and the validation cohort (*P* > 0.05), resulting in a reasonable classification.

**Table 1:** Demographic, clinical, laboratory features

Variable	Total (*n* = 190)	Mild (*n* = 24)	Moderate-to-severed (*n* = 166)	*P*-value	Training cohort (*n* = 134)			Validation cohort (*n* = 56)		
					Mild (*n* = 19)	Moderate-to-severe (*n* = 115)	*P*	Mild (*n* = 5)	Moderate-to-severe (*n* = 51)	P
Birth weight (mean $\pm$ sd)	3.3 $\pm$ 0.5	3.2 $\pm 0.5$	3.3 $\pm 0.5$	0.482	3.2 $\pm$ 0.6	3.3 $\pm 0.5$	0.634	3.3 $\pm$ 0.2	3.4 $\pm 0.5$	0.657
Gestational time, week (mean $\pm$ sd)	39.4 $\pm$ 1.1	39.2 $\pm 1.1$	39.5 $\pm 1.1$	0.207	39.0 $\pm 1.1$	39.5 $\pm 1.1$	0.778	39.9 $\pm 0.8$	39.4 $\pm 1.0$	0.351
Age, day (mean $\pm$ sd)	8.5 $\pm$ 4.7	$7.9\pm 2.1$	8.6 $\pm 4.9$	0.5069	$8.4\pm 2.1$	8.3 $\pm 4.8$	0.936	$6.0\pm 1.2$	$9.5\pm 5.3$	0.182
Gender, n (%)										
F	50 (26.3%)	3 (12.5%)	47 (28.3%)		2 (10.5%)	34 (29.6%)		1 (20.0%)	13 (25.5%)	
M	140 (73.7%)	21 (87.5%)	119 (71.7%)	0.162	17 (89.5%)	81 (70.4)	0.146	4 (80.0%)	38 (74.5%)	1.000
ALT (mean $\pm$ sd)	53.1 $\pm$ 71.7	52.6 $\pm 85.6$	53.2 $\pm$ 69.7	0.968	59.7 $\pm 95.2$	51.8 $\pm 59.7$	0.625	25.2 $\pm 13.0$	56.3 $\pm 88.9$	0.438
AST (mean $\pm$ sd)	117.6 $\pm$ 106.6	$85.4\pm 70.7$	122.3 $\pm 110.2$	0.112	86.7 $\pm 78.1$	125.5 $\pm 112.6$	0.159	77.0 $\pm$ 33.9	115.1 $\pm 105.5$	0.425
Urea nitrogen (mean $\pm$ sd)	5.3 $\pm$ 3.2	4.0 $\pm 1.8$	5.5 $\pm 3.3$	**0.031**	3.9 $\pm 1.8$	5.6 $\pm 3.6$	**0.042**	4.3 $\pm 1.8$	5.2 $\pm 2.6$	0.476
Creatinine (mean $\pm$ sd)	68.1 $\pm$ 36.0	57.5 $\pm 29.8$	69.7 $\pm 36.7$	0.120	53.9 $\pm$ 30.0	71.2 $\pm$ 37.6	0.057	71.2 $\pm 25.7$	66.3 $\pm 34.5$	0.760
CK-MB (mean $\pm$ sd)	129.4 $\pm$ 211.3	119.1 $\pm 202.3$	130.8 $\pm 213.3$	0.800	125.4 $\pm$ 225.2	132.4 $\pm 176.1$	0.878	95.3 $\pm 78.7$	127.3 $\pm 281.4$	0.801
Procalcitonin (mean $\pm$ sd)	8.3 $\pm$ 16.9	6.0 $\pm 17.7$	8.6 $\pm 16.9$	0.478	$2.7\pm 3.6$	9.8 $\pm 19.1$	0.1.06	18.5 $\pm 38.7$	$6.0\pm 9.9$	0.059
Lactic acid (mean $\pm$ sd)	5.8 $\pm 3.1$	5.4 $\pm 2.0$	5.9 $\pm 3.2$	0.453	$5.4\pm 1.9$	6.3 $\pm 3.5$	0.270	5.3 $\pm 2.6$	4.9 $\pm 2.4$	0.774
D-dimer (mean $\pm$ sd)	$4.6\pm 7.6$	2.9 $\pm 2.1$	4.8 $\pm 8.1$	0.243	2.7 $\pm 1.6$	4.8 $\pm 7.9$	0.244	3.7 $\pm 3.7$	4.9 $\pm 8.6$	0.756
CO2 (mean $\pm$ sd)	$37.1\pm$ 13.8	37.8 $\pm 12.5$	37.0 $\pm 14.0$	0.786	37.3 $\pm 13.4$	37.4 $\pm 14.8$	0.963	39.8 $\pm 9.3$	35.9 $\pm 12.0$	0.490
PO2 (mean $\pm$ sd)	79.6 $\pm$ 39.2	68.3 $\pm 18.2$	81.2 $\pm 41.2$	0.133	$66.0\pm 18.7$	76.5 $\pm 27.1$	0.106	77.2 $\pm 14.1$	91.8 $\pm 61.3$	0.600
HCO3-ion (mean $\pm$ sd)	21.0 $\pm 9.1$	25.4 $\pm 5.3$	20.3 $\pm 9.4$	**0.001**	25.5 $\pm 4.7$	20.9 $\pm 9.8$	**0.043**	25.0 $\pm 8.1$	19.1 $\pm 8.3$	0.130
PH (mean $\pm$ sd)	7.3 $\pm$ 0.2	7.4 $\pm 0.1$	7.3 $\pm 0.2$	**0.003**	7.4 $\pm 0.1$	7.3 $\pm 0.2$	**0.005**	7.4 $\pm 0.1$	7.3 $\pm 0.2$	0.321
BE (mean $\pm$ sd)	-4.3 $\pm 9.0$	1.5 $\pm 5.1$	-5.2 $\pm 9.1$	$<0.001$	1.7 $\pm 4.6$	-4.8 $\pm 8.6$	**0.002**	0.70 $\pm 7.4$	-6.0 $\pm 9.6$	0.132

### Univariate and multivariate regression analyses of clinical characteristics

The multivariate regression analysis incorporates all parameters with *P* $<$ 0.05 from the univariate analysis. In the final analysis, urea nitrogen was identified as an independent predictor of moderate-to-severe HIE (Table 2). A clinical model was established by using independent predictors.

**Table 2:** Positive results of univariate and multivariate regression analyses of clinical characteristics

Variable	Univariate regression analysis			
	Odds ration	Lower	Upper	*P*
Urea nitrogen	1.337	1.012	1.765	0.041
PH	0.003	3.401e−05	$0.201$	0.007
BE	0.900	0.838	0.997	0.004

Variable	Multivariate regression analysis		
	Odds ration	CI 95	*P*
Urea nitrogen	1.36	1.02–1.82	0.037

### Radiomics feature selection and construction of Red-score

**Fig. 2:**
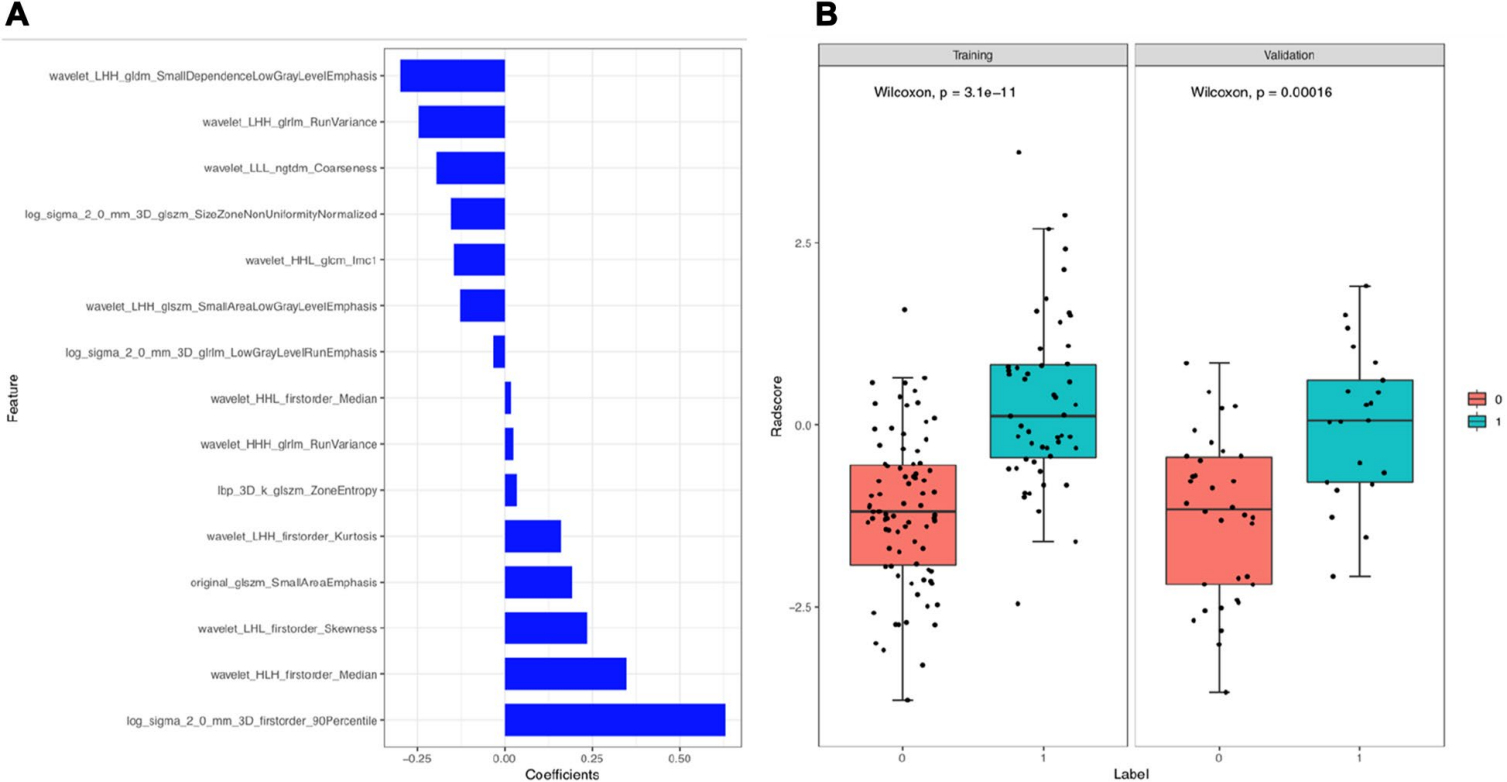
**A** Selection of the most predictive subset of features with the corresponding coefficients after the number of features was determined. **B** Demonstration of significantly higher Rad-scores in the moderate-to-severe group (Label = 1) than those of the mild group (Label = 0), in both the training cohort and the validation cohort

In distinguishing the mild group from moderate-to-severe group, to build the differentiation model, all radiomics features with non-zero coefficients in the LASSO logistic regression model were selected. From the 1316 features in the training cohort, 15 potential predictors were selected after dimensionality reduction (Additional file 3: Fig. S1). Rad-score is a new radiomics signature developed using an equation (Additional file 4: Equation 1). The Wilcoxon test was used to evaluate the difference between the two groups; Fig. 2 shows the distribution of Rad-scores for training and validation cohorts. The moderate-to-severe group had a higher Red-score than the mild group in the training cohort (*P* < 0.001), which was confirmed in the validation cohort (*P* < 0.001).

### Nomogram construction

**Fig. 3:**
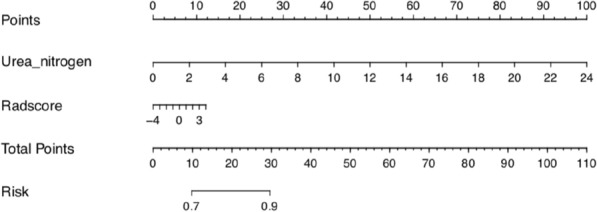
Radiomics nomogram. In the training cohort, the nomogram incorporated the Red-score and Urea nitrogen

Based on the results of univariate and multivariate logistic regression analyses, the independent predictors of clinical characteristics (urea nitrogen) were combined with Red-score to established combined nomogram model (Fig. 3. Additional file 5: Equation 2.)

### Performance and validation of different prediction models

**Fig. 4:**
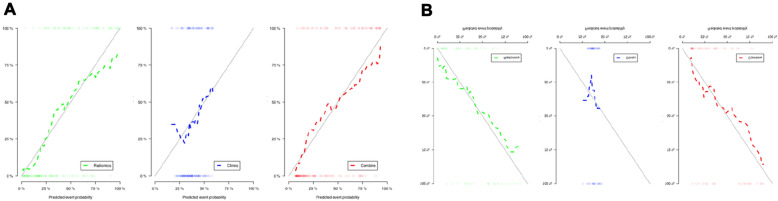
Calibration curves of the three models for the training cohort (**A**) and validation cohort (**B**)

**Fig. 5:**
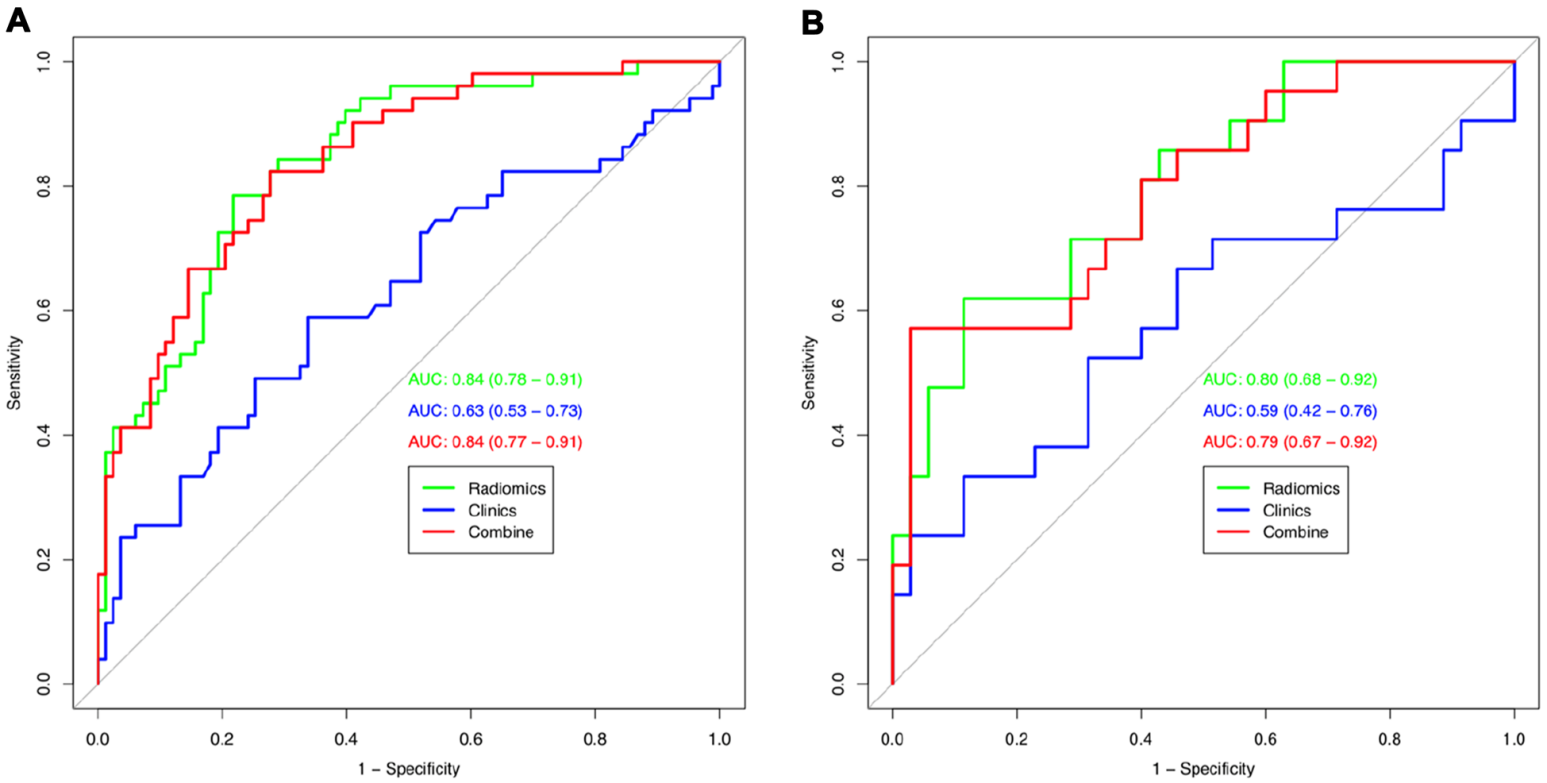
Receiver operating characteristic (ROC) curves for training cohort (**A**) and validation cohort (**B**) for tree models

**Fig. 6:**
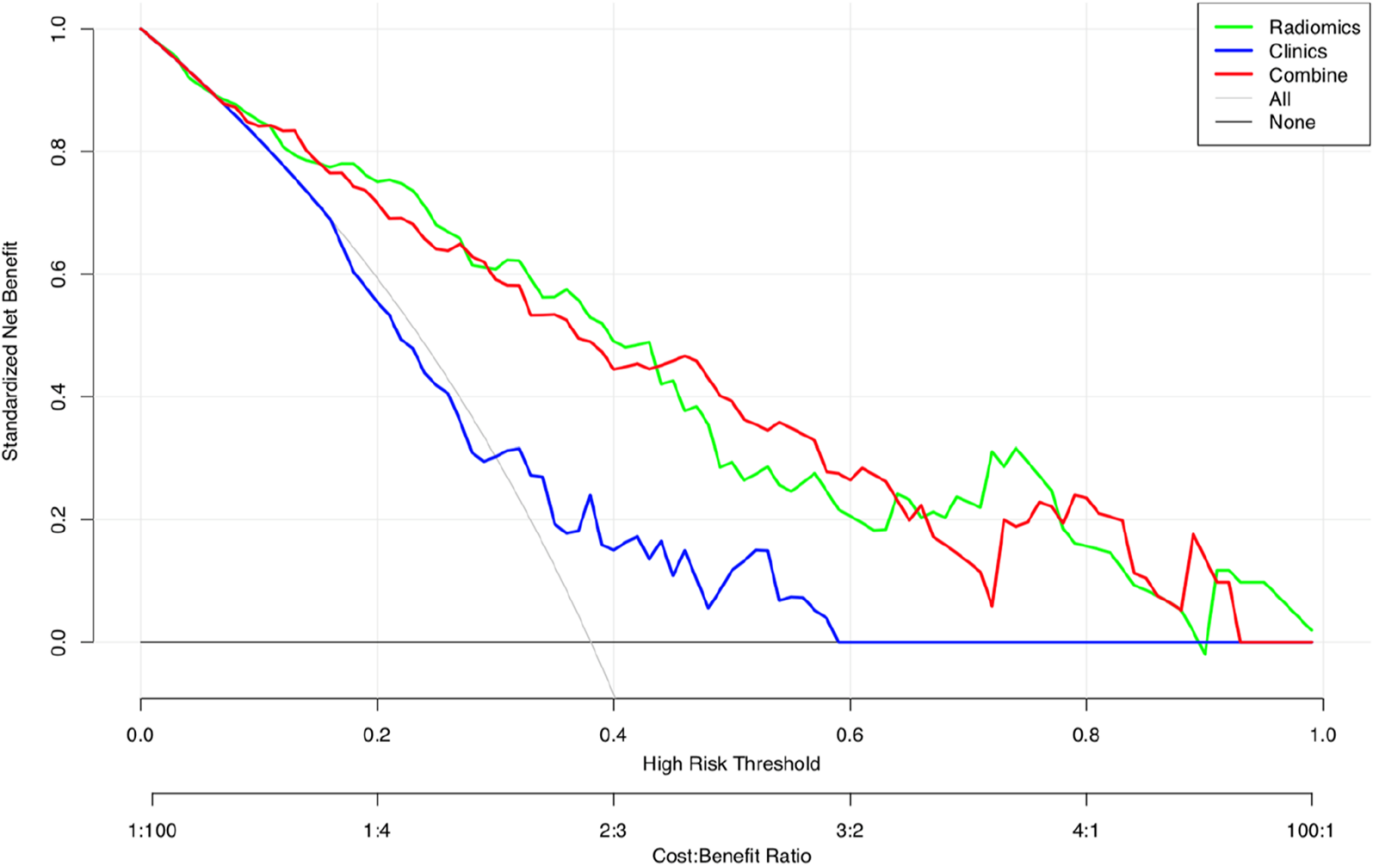
Decision curve analysis for the three models. The green line and red line represent the radiomics model and combined nomogram model, respectively. The blue line represents the clinical model. Decision curves show that radiomics model and combined nomogram model achieved more clinical utility than clinical model

There was no statistically significant difference between calibration curves and ideal curves according to the Hosmer–Lemeshow test, *P* $>$ 0.05 (Fig. 4). According to the training cohort, the AUC of the clinical, radiomics, and combined nomogram models was 0.63, 0.84, and 0.84, respectively. According to the validation cohort, the AUC of the clinical model, radiomics model, and combined nomogram model was 0.59, 0.80, and 0.79, respectively. In the training cohort, there were statistically significant differences in ROC curves between the radiomics and clinical models (*P* = 0.0004538) and between the clinical and combined nomogram model (*P* = 0.0001241). In the validation cohort, there were also significant differences in ROC curves between the radiomics model and the clinical model (*P* = 0.02442) and between the clinical model and the combined nomogram model (*P* = 0.01792). In the validation cohort, there were no significant differences in ROC curves between the radiomics model and the training radiomics model (*P* = 0.7961) and between the radiomics model and the combined nomogram model (*P* = 0.6809). Meanwhile, the radiomics model showed the greatest accuracy (accuracy: 0.784, sensitivity: 0.784, specificity: 0.783, PPV: 0.690, NPV: 0.851.) (Table 3 Fig. 5). Figure 6 shows the DCA based on three models. Decision curves suggest that the use of the combined nomogram model and radiomics model predicts greater benefit in moderathe to severe patients than the use of the clinical model.

**Table 3:** Accuracy and predictive value between three models

	AUC	Accuracy	95% CI	Sensitivity	Specificity	PPV	NPV
Training cohort							
Clinical model	0.63	0.754	0.671–0.824	0.791	0.526	0.910	0.294
Radiomics model	0.84	0.784	0.704–0.850	0.784	0.783	0.690	0.855
Combined model	0.84	0.761	0.680–0.831	0.824	0.723	0.646	0.870
Validation cohort							
Clinical model	0.59	0.750	0.616–0.856	0.784	0.400	0.930	0.154
Radiomics model	0.80	0.678	0.540–0.797	0.620	0.714	0.565	0.758
Combined model	0.79	0.679	0.540–0.797	0.714	0.657	0.556	0.793

### Reproducibility

In radiomics feature extraction, the ICC for inter-observer reproducibility was satisfactory. ICC values of features extracted by observers 1 and 2 in their first extraction ranged from 0.785 to 0.892.

## Discussion

Neonatal HIE is a type of brain injury caused by cerebral blood supply and gas exchange disorders in perinates. In the severe stage, it can lead to irreversible brain injury or even death. The main changes included neurocyte degeneration, necrosis, brain edema, cerebral infarction, and intracranial hemorrhage. Ischemia and hypoxia can cause cerebral vasospasm, increased vascular permeability, and brittleness, and a variety of factors can cause vascular ruptures and hemorrhages [15]. Cerebral hypoxia and ischemia lead to cerebral venous congestion and increased venous pressure, which is easy to cause the expansion of deep cerebral veins and cortical veins in varying degrees [16]. In both healthy and pathologic subjects, SWI can better delineate cerebral venous structures compared to conventional MRI scanning. DMVs drain blood from WM to subependymal veins in the cerebral venous system [17]. Therefore, it is easier to display DMVs area on SWI and obtain ROI more accurately. In this study, we used manual segmentation to extract and select the image radiomics features with predictive values by outlining the ROI of neonatal SWI images.

Using a radiomics model derived from SWI features and deep medullary veins, we demonstrated that moderate-to-severe neonatal HIE could be accurately diagnosed. We observed 15 potential predictors imaging features that were significantly related to it, including wavelet_HLH_firstorder_Median, wavelet_LHH_gldm_SmallDependenceLowGrayLevelEmphasis, and wacelet_LHH. These features have the highest predictive value. A radiomics model was developed and validated for the accurate diagnosis of moderate-to-severe HIE in neonates. In the validation cohort, the AUC of radiomics signatures was 0.80 (95% CI 0.540–0.797), accuracy was 0.687, sensitivity was 0.620, and specificity was 0.714. In the training cohort, the AUC of radiomics signatures was 0.84 (95% CI 0.704–0.850), accuracy was 0.784, sensitivity was 0.784, and specificity was 0.783. As a result, the radiomics model can predict moderate-to-severe neonatal HIE with a certain degree of accuracy. As well, urea nitrogen was the only independent factor that could distinguish mild and moderate-to-severe neonatal HIE in our study. To diagnose moderate-to-severe neonatal HIE accurately, we developed and validated a combined nomogram model. Both the training and validation cohorts of the combined nomogram model did not achieve a higher AUC value than the radiomics model, suggesting the clinical features did not significantly improve the predictive value of radiomics model in distinguishing mild and moderate-to-severe neonatal HIE. Based on DCA results, the combined nomogram and radiomics model performed better than the clinical model for moderate-to-severe neonatal HIE prediction. Additionally, the training cohort and validation cohort calibration curves were constructed. According to the calibration curves based on the validation cohort, the proposed models have a favorable classification performance.

Some studies used MRI score ability to detect HIE abnormalities, although conventional and further MRI techniques have described HIE features in neonates with HIE [18–20]; our study is the first article that uses a nomogram model to distinguish mild HIE form moderate-to-severe HIE. In the past, there were few studies on the use of radiomics to analysis neonatal HIE. In previous studies, infants with ischemic injuries can be differentiated based on texture features. Weiss et al. are working on combining radiomics with machine learning to detect lesions and predict outcomes in patients with HIE; however, they have not published their findings at the time of writing [21]. Kim et al. [12] only used histogram analysis to study the feasibility in infants with ischemic injury, and their study only used a limited number of infants. Their AUC was 0.865, which was similar to our study. Based on the texture of the basal ganglia and thalami in neonates, Fatma et al. [13] demonstrated accurate diagnosis of moderate-to-severe HIE, with a sensitivity of 95% and accuracy of 94.3%. However, it is difficult to directly compare the predictive power of our model to Fatma et al. models. In this model, previous studies only used texture features. Texture features are very useful for identifying target images with obvious texture features; however, its main disadvantage is that when the resolution of the image and the illumination of the target change, the texture of the target image may produce a large deviation and affect the classification effect. A larger sample size of 170 patients was used in our study, compared to a sample size ranging from 7 to 35 patients in previous studies. A small sample size will lead to overfitting and affect the authenticity of the data. In our study, we first added clinical independent factor combined radiomics features to establish a nomogram model. The nomogram model visualizes the radiomics features and clinical predictors and provides a simple and easy-to-use tool for the individualized prediction of HIE in neonates and predict the severity of HIE.

Despite a lack of understanding about the underlying mechanism for radiomics and nomograms that reflect HIE stages, we speculate that radiomics and nomogram can reveal the micro-changes in hypoxic injury. A wide variety of pediatric neurological disorders have been studied using SWI in previous studies, including chronic ischemia, developmental venous anomalies, microhemorrhages, convulsive disorder, and hypoxic-ischemic injury [5, 22, 23]. For ischemic changes, SWI can clearly show the abnormal dilation of small veins. In the early stage of HIE, brain histopathology shows cerebral edema and small vein hyperemia and dilation. After cerebral hypoxia, small artery reactive dilation and low brain oxygen uptake rate resulted in hemodynamic and blood compensatory damage at the blood level of vascular tissue. This led to an increase in the proportion of small vein deoxyhemoglobin, which was shown as small vein dilation on SWI [24, 25]. Kitamura et al. have suggested that the degree of deep medullary vein dilatation in children with hypoxic-ischemic brain injuries can indicate the prognosis of the nervous system.

There are several limitations to this study. First, it is a retrospective study done by a single center with no external validation; there can also be a limited generalizability due to case selection bias. Second, although this study included a relatively large number of neonates, the cohort was still small when compared with other radiomics studies, especially the mild HIE group; our results may not be generalizable due to these factors. To validate our findings, we need a large-scale, prospective, multicenter study. Thirdly, as we know, in neonates with perinatal asphyxia, the combination of T1WI, T2WI, and DWI was the most commonly used method to detect cerebral damage [26]. To enhance the clinical impact of these models, we need to investigate whether they can further improve diagnostic efficiency of HIE severity, and whether they can predict neurodevelopment outcomes in HIE along with T1WI, T2WI, and DWI. Fourth, manual segmentation was used to delineate the ROI of the DMVs, but automatic or semi-automatic segmentation was not used for comparison and verification, which had a certain subjective impact. Finally, a severe perinatal asphyxia also affects the cerebral cortex, basal ganglia, and thalami [27], and was not considered in this study because the discrimination of the ROI in those areas would not be reliable on SWI. The above deficiencies need to be further improved by further research.

## Conclusion

For the classification of mild HIE and moderate-to-severe HIE in neonates, the combined nomogram model and radiomics model can be reliable and effective. Even if there is no visually detectable difference between the DMVs on SWI, they may generate objective features which can indicate differences. In our study, we suggest that radiomics analysis of SWI can be a useful tool in predicting the severity of brain injury of infants with HIE.

## Supplementary Information

**Additional file 1.** S1 Imaging Procesess.

**Additional file 2.** S2 Image Segmentation and Radiomics feature extraction.

**Additional file 3.** Figure S1 The least absolute shrinkage and selection operator (LASSO) including the selection of the regular parameter λ and determination of the number of features.

**Additional file 4.** Equation S 1 Rad-score formula.

**Additional file 5.** Equation S 2 Nomoscore formula.

## Data Availability

All data generated or analyzed during this study are included in this published article.

## Abbreviations

AUC: Area under the curve
BE: Base excess
DCA: Decision curve analysis
DICOM: Digital Imaging and Communication in Medicine
DMV: Deep medullary veins
GLCM: Gray-level co-occurrence matrix
GLDM: Gray-level dependence matrix
GLRLM: Gray-level run-length matrix
GLSZM: Gray-level size-zone matrix
HIE: Hypoxic-ischemic encephalopathy
ICC: Intraclass correlation coefficient
LASSO: Least absolute shrinkage and selection operator
LBP: Local binary mode
LoG: Logarithmic transformation
MRI: Magnetic resonance imaging
mRMR: Minimum redundancy maximum relevance
NGTDM: Neighborhood gray difference matrix
PACS: Picture archiving and communication system
ROC: Receiver operating characteristic
ROI: Regions of interest
SWI: Susceptibility-weighted imaging
WM: White matter
