# Supplementary material for: Radiomics based of deep medullary veins on susceptibility-weighted imaging in infants: predicting the severity of brain injury of neonates with perinatal asphyxia
Source: Eur J Med Res. 2023 Jan 6;28:9. doi: 10.1186/s40001-022-00954-y (PMC9817267; doi:10.1186/s40001-022-00954-y)
Supplement: Supplementary file 1 — Additional file 1. S1 Imaging Procesess. [file 40001_2022_954_MOESM1_ESM.docx]

**Additional File 1**

**S1 Imaging Procesess**

The imaging process was included: First, to eliminate the intrinsic dependency on voxel size for the radiomic features, a resampling method with a linear interpolation algorithm was used to normalize the voxel resolution. Higher-order texture analysis features were derived from different directions and different scales, so the anisotropic voxels scanned at other size were resampled to form voxels, i.e, 0.750 mm* 0.750 mm* 0.750 mm. Second, a Gaussian filter was used to remove “unwanted signals”. Because different scanners had the same gray level, gray level normalization was not performed here.
