# Supplementary material for: Radiomics based of deep medullary veins on susceptibility-weighted imaging in infants: predicting the severity of brain injury of neonates with perinatal asphyxia
Source: Eur J Med Res. 2023 Jan 6;28:9. doi: 10.1186/s40001-022-00954-y (PMC9817267; doi:10.1186/s40001-022-00954-y)
Supplement: Supplementary file 2 — Additional file 2. S2 Image Segmentation and Radiomics feature extraction. [file 40001_2022_954_MOESM2_ESM.docx]

**Additional File 2**

**S2 Image Segmentation and Radiomics feature extraction**

T1WI, T2WI, ADC and SWI images for each patient were reviewed independently by two pediatric radiologists (observer 1, Z.X.M, a 5-year-experienced pediatric radiologist and observer 2, Y.Y, a 10-year-experienced pediatric radiologist) with anonymized clinical data. Any discrepancies between the two radiologists were settled by a third pediatric radiologist (L.X.M, a 25-year-experienced pediatric radiologist). In this study, DMVs were assessed and quantified with created region of interests (ROIs), closed to the lateral ventricles [1]. ROIs were drawn at the right and left WM involving the DMV. We selected two axial slices to draw the ROI on the level where the DMV showed its typical fan pattern of drainage into the subependymal vein. When drawing the ROIs, we excluded subependymal veins and large cortical veins. Then, we used AK software (Artificial Intelligence Kit v.3.3.0, GE Healthcare) to extract radiomics features. The features included first order, shape (Shape), gray-level run-length matrix (GLRLM), gray-level co-occurrence matrix (GLCM), gray-level dependence matrix (GLDM), gray-level size-zone matrix (GLSZM), and neighborhood gray difference matrix (NGTDM). The selected image transformations were: wavelet transformation (Wavelet), Level 1; logarithmic transformation (LoG), parameter Sigma selection 2.0, 3.0; local binary mode (LBP), Level 2, Radius 1.0, Subdivision select 1. A total of 1,316 features were extracted.

**Reference:**

[1] Kuijf HJ, Bouvy WH, Zwanenburg JJ, et al. Quantification of deep medullary veins at 7 T brain MRI. Eur Radiol. 2016;26(10):3412–8.
