# Supplementary material for: Radiomics based of deep medullary veins on susceptibility-weighted imaging in infants: predicting the severity of brain injury of neonates with perinatal asphyxia
Source: Eur J Med Res. 2023 Jan 6;28:9. doi: 10.1186/s40001-022-00954-y (PMC9817267; doi:10.1186/s40001-022-00954-y)

**Additional File 3**

**Figure S1** The least absolute shrinkage and selection operator (LASSO) including the selection of the regular parameter \(\lambda\) and determination of the number of features.


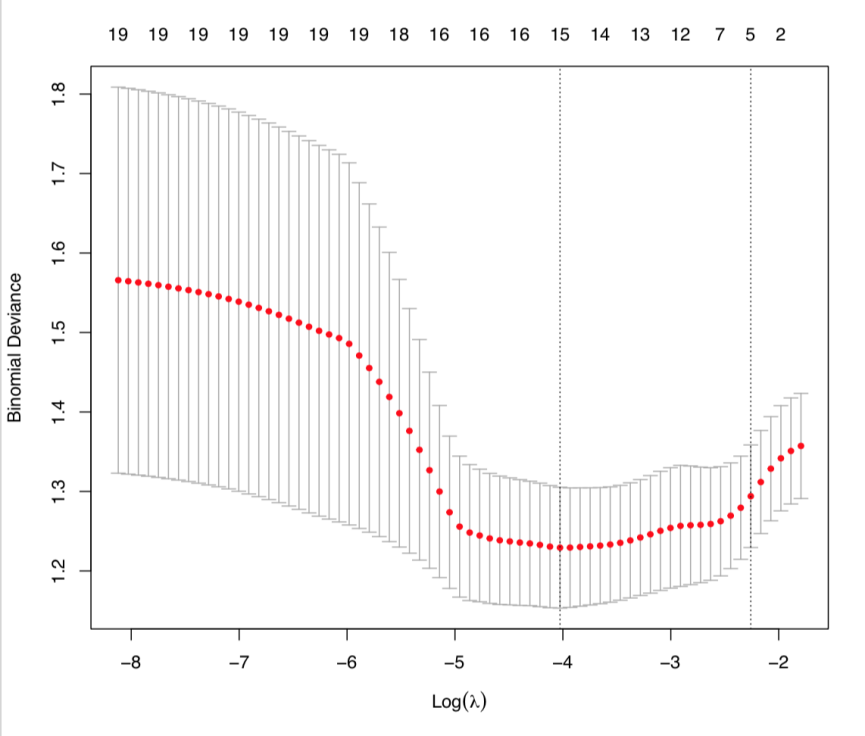


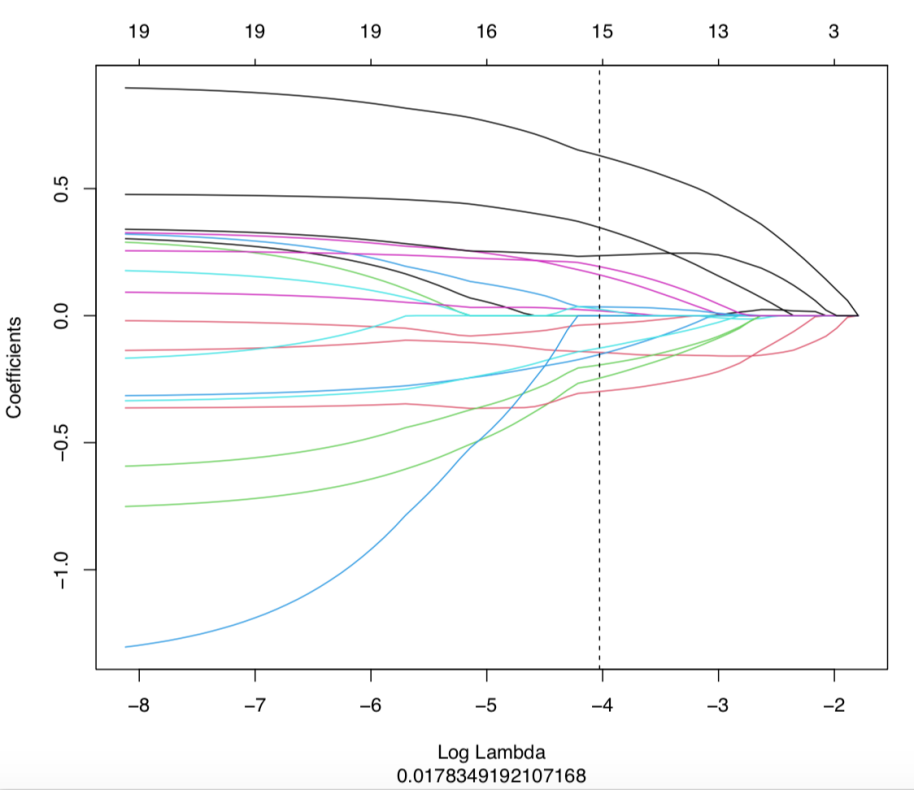

Supplement: Supplementary file 3 — Additional file 3. Figure S1 The least absolute shrinkage and selection operator (LASSO) including the selection of the regular parameter λ and determination of the number of features. [file 40001_2022_954_MOESM3_ESM.docx]
