# Supplementary material for: Radiomics based of deep medullary veins on susceptibility-weighted imaging in infants: predicting the severity of brain injury of neonates with perinatal asphyxia
Source: Eur J Med Res. 2023 Jan 6;28:9. doi: 10.1186/s40001-022-00954-y (PMC9817267; doi:10.1186/s40001-022-00954-y)
Supplement: Supplementary file 4 — Additional file 4. Equation S 1 Rad-score formula. [file 40001_2022_954_MOESM4_ESM.docx]

**Additional File 4**

**Equation S 1**

Rad-score formula

"radscore=0.629825973428418*log_sigma_2_0_mm_3D_firstorder_90Percentile+0.346484769778514*wavelet_HLH_firstorder_Median+0.235842106794395*wavelet_LHL_firstorder_Skewness+0.192932857181591*original_glszm_SmallAreaEmphasis+0.160504078436445*wavelet_LHH_firstorder_Kurtosis+0.0343670169425521*lbp_3D_k_glszm_ZoneEntropy+0.0251172765169056*wavelet_HHH_glrlm_RunVariance+0.0187560922061842*wavelet_HHL_firstorder_Median+-0.0335023890741496*log_sigma_2_0_mm_3D_glrlm_LowGrayLevelRunEmphasis+-0.127906576817766*wavelet_LHH_glszm_SmallAreaLowGrayLevelEmphasis+-0.144746396058406*wavelet_HHL_glcm_Imc1+-0.152855654858139*log_sigma_2_0_mm_3D_glszm_SizeZoneNonUniformityNormalized+-0.194134035522207*wavelet_LLL_ngtdm_Coarseness+-0.246031895063348*wavelet_LHH_glrlm_RunVariance+-0.298628672960307*wavelet_LHH_gldm_SmallDependenceLowGrayLevelEmphasis+-0.683688750272229*(Intercept)"
