# Supplementary material for: Radiomics based of deep medullary veins on susceptibility-weighted imaging in infants: predicting the severity of brain injury of neonates with perinatal asphyxia
Source: Eur J Med Res. 2023 Jan 6;28:9. doi: 10.1186/s40001-022-00954-y (PMC9817267; doi:10.1186/s40001-022-00954-y)
Supplement: Supplementary file 5 — Additional file 5. Equation S 2 Nomoscore formula. [file 40001_2022_954_MOESM5_ESM.docx]

**Additional File 5**

**Equation S 2**

Nomoscore formula

"nomoscore=0.5966411737973*(Intercept)+0.283652825854209*Urea_nitrogen+0.103365987824817*Radscore"
